# Maternal diet affects juvenile Carpetan rock lizard performance and personality

**DOI:** 10.1002/ece3.5882

**Published:** 2019-12-09

**Authors:** Gergely Horváth, Gonzalo Rodríguez‐Ruiz, José Martín, Pilar López, Gábor Herczeg

**Affiliations:** ^1^ Behavioural Ecology Group Department of Systematic Zoology and Ecology Eötvös Loránd University Budapest Hungary; ^2^ Department of Evolutionary Ecology Museo Nacional de Ciencias Naturales CSIC Madrid Spain

**Keywords:** animal personality, behavioral predictability, corticosterone, individual state, maternal diet

## Abstract

Differences in both stable and labile state variables are known to affect the emergence and maintenance of consistent interindividual behavioral variation (animal personality or behavioral syndrome), especially when experienced early in life. Variation in environmental conditions experienced by gestating mothers (viz. nongenetic maternal effects) is known to have significant impact on offspring condition and behavior; yet, their effect on behavioral consistency is not clear. Here, by applying an orthogonal experimental design, we aimed to study whether increased vitamin D_3_ content in maternal diet during gestation (vitamin‐supplemented vs. vitamin control treatments) combined with corticosterone treatment (corticosterone‐treated vs. corticosterone control treatments) applied on freshly hatched juveniles had an effect on individual state and behavioral consistency of juvenile Carpetan rock lizards (*Iberolacerta cyreni*). We tested the effect of our treatments on (a) climbing speed and the following levels of behavioral variation, (b) strength of animal personality (behavioral repeatability), (c) behavioral type (individual mean behavior), and (d) behavioral predictability (within‐individual behavioral variation unrelated to environmental change). We found higher locomotor performance of juveniles from the vitamin‐supplemented group (42.4% increase), irrespective of corticosterone treatment. While activity personality was present in all treatments, shelter use personality was present only in the vitamin‐supplemented × corticosterone‐treated treatment and risk‐taking personality was present in corticosterone control treatments. Contrary to our expectations, behavioral type was not affected by our treatments, indicating that individual quality can affect behavioral strategies without affecting group‐level mean behavior. Behavioral predictability decreased in individuals with low climbing speed, which could be interpreted as a form of antipredator strategy. Our results clearly demonstrate that maternal diet and corticosterone treatment have the potential to induce or hamper between‐individual variation in different components of boldness, often in interactions.

## Graphical abstract

Individual behavioral strategy is a complex phenotypic trait with hierarchically structured components. Here, by using an orthogonal experimental design, we show that maternal diet and corticosterone treatment have the potential to induce or erode among‐individual variation in different components of boldness of juvenile Carpetan rock lizards, often in interactions. Considering that natural selection can only operate on existing among‐individual phenotypic variation, our results imply that short‐term differences in environmental conditions and individual state might—at least temporarily—release a population from natural selection acting on behavioral variation.

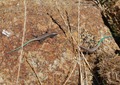

## 1 INTRODUCTION

Behavior is a highly flexible phenotypic trait (West‐Eberhard, 2003); yet, consistent differences between individuals over time and situations within a single (animal personality) and across multiple (behavioral syndrome) behaviors are often traceable (Bell, Hankison, & Laskowski, 2009; Garamszegi, Markó, & Herczeg, 2012; Sih, Bell, Johnson, & Ziemba, 2004). Although “animal personality research” is one of the fastest developing fields of behavioral ecology, studies on the evolutionary importance of within‐individual behavioral variability started to accumulate only recently (Araya‐Ajoy & Dingemanse, 2017; Dingemanse, Kazem, Réale, & Wright, 2010; Stamps, Briffa, & Biro, 2012). Several studies demonstrated lately that components of within‐individual variation, known as behavioral plasticity (behavioral response induced by environmental change) and behavioral predictability (behavioral variation independent from environmental change), are not only integrated into an individual's behavioral strategy (Chang, Teo, Norma‐Rashid, & Li, 2017; Lichtenstein, Chism, Kamath, & Pruitt, 2017; Urszán et al., 2018; Velasque & Briffa, 2016), but individual‐specific variation in these components exists (Biro & Adriaenssens, 2013; Briffa, 2013; Briffa, Bridger, & Biro, 2013; Ioannou & Dall, 2016; Westneat, Wright, & Dingemanse, 2015). Thus, plasticity and predictability of individual behavior are potentially independent traits, which may be targeted by selection directly (Brembs, 2011; Mathot & Dingemanse, 2014).

In behavioral ecology, “state” is defined as every characteristic of an individual, which affects the costs and benefits of behavioral decisions (see Houston & McNamara, 1999; Sih et al., 2015). Among the standard theories interpreting the emergence of interindividual behavioral variation, state dependence implies the role of individual state differences in creating behavioral consistencies (Bijleveld et al., 2014; Dall, Houston, & McNamara, 2004; Rands, Cowlishaw, Pettifor, Rowcliffe, & Johnstone, 2003; Sih et al., 2015). Recent empirical data suggest that not only inherently stable (e.g., gender, morphology; see Sih et al., 2015), but even labile state variables and/or short‐term differences in environmental conditions can shape behavioral consistency (DiRienzo, Niemelä, Hedrick, & Kortet, 2016; Horváth, Martín, López, Garamszegi, & Herczeg, 2017; Horváth, Mészáros, et al., 2017; Lichtenstein et al., 2016; Urszán, Garamszegi, et al., 2015; Urszán et al., 2018; Urszán, Török, Hettyey, Garamszegi, & Herczeg, 2015). Such variation can have particularly strong effects during early stages of ontogeny (Dingemanse et al., 2007; DiRienzo & Montiglio, 2016; Urszán et al., 2018), but they are also expected to create long‐lasting effects (DiRienzo, Niemelä, Skog, Vainikka, & Kortet, 2015; DiRienzo, Pruitt, & Hedrick, 2012; Krause, Krüger, & Schielzeth, 2017).

In amniotes, environmental differences experienced by the mother (i.e., nongenetic maternal effects; see Roff, 1998) may result in considerable and permanent differences in the offspring's physiology, morphology, and behavior (Ensminger, Langkilde, Owen, MacLeod, & Sheriff, 2018; Gosling, 2008; Munch et al., 2018; Räsänen & Kruuk, 2007). Although several studies suggest that maternal effects could drive the emergence of behavioral consistency (Ghio, Leblanc, Audet, & Aubin‐Horth, 2016; Langenhof & Komdeur, 2018; McCormick, 2006; Reddon, 2012; Rokka, Pihlaja, Siitari, & Soulsbury, 2014), data on this topic are rather contradictory (Arnaud et al., 2017; Hinde et al., 2015; Petelle, Dang, & Blumstein, 2017; Ruuskanen, 2018). Provision to the egg can be seen as a specific dimension of maternal effects (Deeming & Ferguson, 1991; Stewart & Ecay, 2010), as in oviparous vertebrates, such as many reptiles, trace nutrients (vitamins and minerals) must be deposited into the yolk during oogenesis (Deeming & Ferguson, 1991). In many cases, binding proteins responsible for the delivery of certain vitamins can be influenced by the availability of the vitamin itself; thus, the amount of certain vitamins in the yolk and consequently the future state of the offspring highly depend on the composition of maternal diet. Hence, dietary intake of vitamins during gestation is expected to directly affect the state of offspring, and eventually, to affect the emergence of behavioral consistency in juveniles.

In contrast to maternal effects, the relationship between glucocorticoid hormones and personality was studied frequently (Carere, Caramaschi, & Fawcett, 2010; Ghio et al., 2016; Koolhaas et al., 1999; Medina‐García, Jawor, & Wright, 2017; Mell et al., 2016; Muraco, Aspbury, & Gabor, 2014; Øverli et al., 2007). Glucocorticoids are metabolic hormones with numerous pleiotropic effects (MacDougall‐Shackleton, Bonier, Romero, & Moore, 2019), they play a primary role in energy mobilization and facilitate behavioral and physiological shifts to minimize the effect of stress (Cote, Clobert, Meylan, & Fitze, 2006; Cote, Clobert, Montes Poloni, Haussy, & Meylan, 2010; Romero & Wingfield, 2015). Corticosterone is an important glucocorticoid in vertebrates, being part of the complex physiological stress response and promoting behaviors that directly or indirectly enhance survival (e.g., decreased reproductive activity [Belliure, Smith, & Sorci, 2004], increased locomotor activity [Clobert et al., 2000]). Experimental data (mainly on avian species) indicate that stress‐induced corticosterone affects cognitive abilities (Bebus, Small, Jones, Elderbrock, & Schoech, 2016; Bókony et al., 2014; Jones, Bebus, Ferguson, Bateman, & Schoech, 2016; Ruiz‐Gomez, Huntingford, Øverli, Thörnqvist, & Höglund, 2011). In addition, bold behavioral types (i.e., risk‐prone, active, superficial explorer) seem to be associated with low, while shy behavioral types (i.e., risk‐averse, less active, thorough explorer) with high basal levels of corticosterone (see Carere et al., 2010; Cockrem, 2007; Koolhaas et al., 1999). On the other hand, this pattern is not general (Koolhaas et al., 1999); moreover, there is no direct link between stress‐induced corticosterone level and personality in some cases (Garamszegi, Rosivall, et al., 2012; Medina‐García et al., 2017). Hence, the exact role of corticosterone in the emergence of behavioral consistency is still unclear.

Here, we aimed to study how maternal diet and corticosterone treatment during early stages of life affect behavioral consistency of juvenile Carpetan rock lizards (*Iberolacerta cyreni*). As *I. cyreni* became one of the best studied reptiles during the past decades (Horváth et al., 2016; Horváth, Martín, et al., 2017; Martín & López, 2000, 2006; Martín & Salvador, 1993), it is a suitable model for our purposes, because the results can be interpreted in a broad eco‐evolutionary context. To this end, we applied an orthogonal experimental design: (a) Gravid mothers were supplemented with either vitamin D_3_ or sunflower oil control and (b) juveniles from each litter were randomly assigned to either corticosterone treatment or soybean oil control treatment. Vitamin D_3_ concentration and binding proteins identical to that of the avian yolk are evidently present in several reptile species (Deeming & Ferguson, 1991; Laing & Fraser, 1999); moreover, calcium transport during late stages of embryogenesis is found to be highly sensitive to dietary intake of vitamin D_3_ in domestic fowls (Stewart & Ecay, 2010; Tuan & Suyama, 1996). Hence, maternal vitamin D_3_ supplementation potentially induces substantial differences in the offspring' permanent state, in particular, in locomotor performance, which is a suitable proxy of fitness (Irschick, Herrel, Vanhooydonck, Huyghe, & Van Damme, 2005; Winchell, Maayan, Fredette, & Revell, 2018). Therefore, offspring of vitamin‐supplemented *I. cyreni* mothers are expected to be of higher quality (see Bergeron, Baeta, Pelletier, Réale, & Garant, 2011; Wilson & Nussey, 2010). In contrast, corticosterone treatment can be considered as an acute manipulation of offspring state, resulting in short‐term differences. We measured climbing speed (as a measure of locomotor performance) of the juveniles. Then, we repeatedly assayed three behaviors (activity, refuge use, risk‐taking). We tested for treatment effects on (a) the presence/absence/strength of animal personality (behavioral repeatability), (b) behavioral type (individual mean behavior), and (c) behavioral predictability (within‐individual behavioral variation unrelated to environmental change). We note here that our assays testing animal personality during a short time period do not represent personality in the classical sense; however, rapid assays combined with relatively long acclimation periods potentially provide sufficient estimates on repeatability (Biro, 2012). We hypothesized that our treatments will have strong effects on physiological processes, and consequently, on performance and behavior. We expected higher climbing speed in lizards with vitamin‐supplemented mothers. Based on our previous and other empirical results (see Horváth, Martín, et al., 2017; Horváth, Mészáros, et al., 2017; Lichtenstein et al., 2016), we predicted that animal personality will be stronger in optimal (vitamin‐supplemented mother × corticosterone control) rather than suboptimal (vitamin control mother × corticosterone‐treated) treatments, because optimal conditions might favor the emergence of individual strategies. We were also interested to see whether increased variation in individual states would increase behavioral variation. We tested this question by comparing the strength of personality between the pooled sample (high variation) and the different treatment groups (low variation). We also predicted increased boldness in general for juveniles from vitamin‐supplemented mothers, following the state‐dependent safety principle (see Dingemanse & Wolf, 2010; Sih et al., 2015) and decreased behavioral activity in corticosterone‐treated individuals. Regarding behavioral predictability, two different scenarios are plausible. Recent studies suggest that higher‐state individuals are less predictable (DiRienzo & Montiglio, 2016; Lichtenstein et al., 2017); thus, lizards from vitamin‐supplemented mothers could be expected to be less predictable. However, it is equally plausible that low‐quality individuals may need to be unpredictable to avoid predation. We formed no explicit predictions regarding treatment interactions, but expected milder effects of corticosterone treatment on individuals of higher quality.

## 2 MATERIALS AND METHODS

### 2.1 Collection and housing of females

We noosed 17 gravid females between 20 May and 21 June 2016 at the Puerto de Navacerrada (Sierra de Guadarrama Mts., Community of Madrid, Spain, 1,900 m asl, approximately). This habitat is characterized by a large cover of granite rocks and dense vegetation of *Cytisus* and *Juniperus* shrubs. Gravid *I. cyreni* females were identified in the field by the presence of bite marks made by males during copulation. Animals were transported to the “El Ventorrillo” field station of the Museo Nacional de Ciencias Naturales, ca. 5 km from the capture site, where they were housed outdoor, individually in gray opaque boxes (57 cm × 37 cm × 30 cm; length, width, height, respectively). In the boxes, we provided a layer of coconut fiber (2–3 cm, approximately) as substrate and a hollow brick as shelter. During the whole captivity period (including treatments, see below), food (crickets [*Achaeta domestica*] and mealworms [*Tenebrio molitor*]) and water were provided ad libitum. All females were released at their original capture points, without any sign of injury, a few days after oviposition.

### 2.2 Maternal supplementation treatment

Females were randomly assigned to vitamin D_3_ supplementation treatment (*N* = 7; hereafter vitamin‐supplemented) and control treatment (*N* = 10; hereafter vitamin control). Supplemented females were fed daily with a dietary dose of 0.20 μg (=8 IU) vitamin D_3_ (MYPROTEIN, The Hut.com Ltd.) diluted in 0.25 ml sunflower oil (Coosol, Coosol S.L.). Treatments lasted until oviposition, which varied by (minimum) 20 to (maximum) 25 days. To ensure that all females ingested the same amount of vitamin solution, lizards were gently handled and a sterile syringe with a cannula was used to slowly deliver the solution into their mouth. Lizards were released back to their cages when it was ensured that they had swallowed the entire dose. Previous work from Langkilde and Shine (2006) suggests that simple handling of a similarly sized lizard is not particularly stressful; moreover, plasma corticosterone levels recover to background level relatively quick. As handling related to our experimental procedure took only a few minutes daily, we are convinced that this treatment did not cause significant and prolonged stress in female *I. cyreni*. Control females were treated in the same way (including handling), but they were provided with 0.25 ml of sunflower oil alone, without the vitamin D_3_ solution. Note that sunflower oil contains no vitamin D_3_.

### 2.3 Offspring characteristics and corticosterone treatment

Upon oviposition, eggs were collected and individually relocated into 170‐ml plastic jars. The jars were filled with moistened perlite (1:1 perlite–water ratio) and covered with a lid in order to maintain humidity. Eggs were incubated at 27.5°C (mean incubation time ± *SD*: 37 ± 2 days) using air incubators (FRIOCELL FC‐B2V‐M/FC404, MMM Medcenter GmbH). Seventeen females laid 100 eggs; clutch size varied between four and nine (mean clutch size ± *SD*: 5.64 ± 1.68 eggs). Eighty‐six juveniles hatched altogether (mean number of offspring per clutch ± *SD*: 4.16 ± 2.19). Once the juveniles were born, we measured snout‐vent length (SVL) with a digital caliper (to the nearest 0.01 mm). Body mass (BM) was measured using a digital scale (to the nearest 0.001 g). As all offspring retain hemipenes well after birth, sex determination was not possible, and thus, we did not consider offspring sex in our analyses. We did not test for potential intraclutch differences in resource allocation, as this requires performing various invasive techniques on eggs (see Poisbleau et al., 2013; Warner, Bonnet, Hobson, & Shine, 2008).

Juveniles were housed individually in plastic boxes (17 cm × 17 cm × 9 cm; length, width, height, respectively) under laboratory indoor conditions. A thin layer of coconut fiber was used as substrate, and a piece of tile as shelter. Photoperiod (12 hr light:12 hr dark; natural range in September at the habitat) was provided by Repti Glo 5.0 Full Spectrum Terrarium Lamps (Exo Terra, Rolf V. Hagen Inc.). In the daytime, thermoregulation was provided by 80 W Solar GLO heat bulbs (Exo Terra, Rolf V. Hagen Inc.), average substrate temperature beneath the lamps was 27.57°C (±2.38°C [standard deviation, *SD*]). Temperature decreased substantially during the night (ca. 20°C), imitating the natural conditions of the species during early autumn. Two juveniles from each clutch were chosen randomly and then assigned randomly to the corticosterone‐treated or corticosterone control treatments (sample sizes for the different treatment combinations: vitamin‐supplemented × corticosterone‐treated = 7; vitamin supplemented × corticosterone control = 7; vitamin control × corticosterone‐treated = 10; vitamin control × corticosterone control = 10). Juveniles in the corticosterone group were dorsally administered (with a pipette) with 0.5 μl of a corticosterone solution (3 μg corticosterone/1 μl soybean oil), while in the control group with 0.5 μl of soybean oil. These volumes and concentrations are identical to those used in previous experiments using offspring of Common lizard (*Zootoca vivipara*), a species of similar size, and known to elevate plasma corticosterone levels in various, small‐sized reptile species (Cadby, Jones, & Wapstra, 2010; Meylan, Belliure, Clobert, & Fraipont, 2002). Treatments were applied every day at 18.00 hr (UTC + 02.00) for 10 days between September 03 and 12. During habituation, treatments and behavioral assays (see below), water and food (*Acheta domestica*) were provided ad libitum.

### 2.4 Behavioral assays

**Figure 1:**
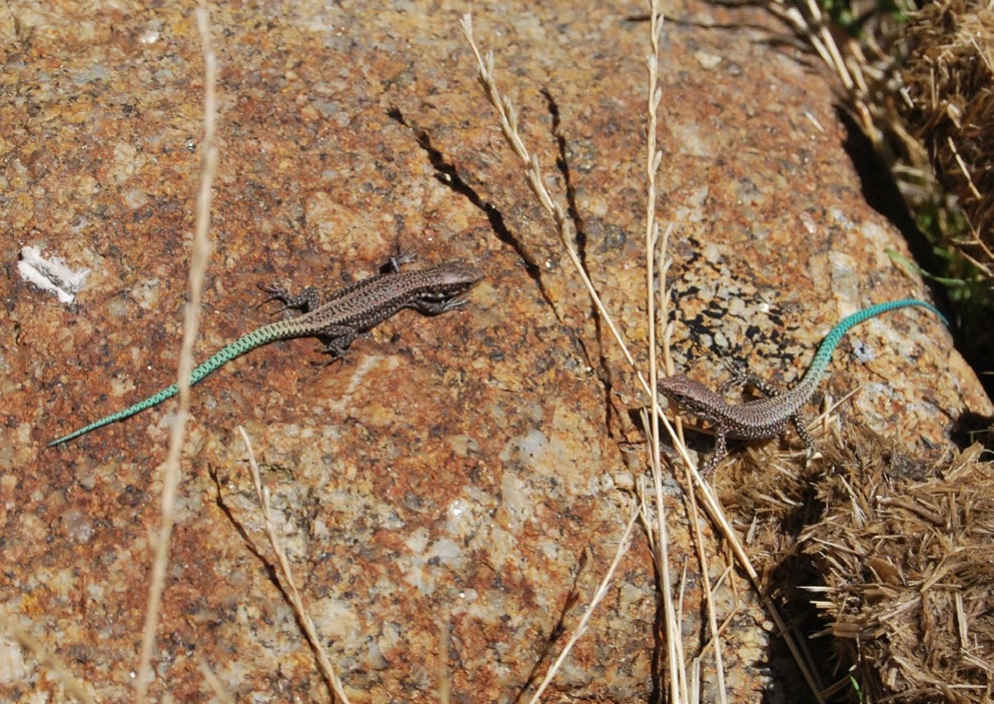
Juvenile Carpetan rock lizards (*Iberolacerta cyreni*). Photograph by Gonzalo Rodríguez‐Ruiz

Activity, shelter use, and risk‐taking of 1‐ to 2‐week‐old juveniles (Figure 1) were tested four times between 06 and 13 September starting three days after the start of treatments. We observed risk‐taking on every second day to provide time to animals to recover from the handling related to this assay. Activity of juveniles was video‐recorded between 10.00 and 11.00 hr (UTC + 02.00) using video cameras (Panasonic HC‐V160, Panasonic Co.). We assessed movement activity (hereafter activity) of animals in their home boxes based on a 30‐min sample from every 1 hr video footage (sum of three 10‐min intervals, distributed equally: 5–15, 25–35, 45–55 min). The program MotionMeerkat (Weinstein, 2015) was used to evaluate the time (s) the animals spent moving. We also evaluated the time (s) the animals spent under the shelter during the observation period (hereafter shelter use) to get another measure of activity (Réale, Reader, Sol, McDougall, & Dingemanse, 2007).

Risk‐taking was evaluated between 14.00 and 15.00 in unfamiliar, hence potentially risky environments. Nine arenas (white plastic boxes; 17 cm × 17 cm × 9 cm; length, width, height, respectively) were used, and tests were performed in four rounds on each day, to assay all juveniles once in every test day. Arenas were thoroughly washed using detergent between assays to remove chemical stimuli that may have been left by the last individual. The order and placement of the animals were randomized within day. First, the experimenter caught the juvenile and dropped it into the test arena from a standardized height of 10 cm. This was done to mimic a situation when the individual was caught by a predator but managed to escape. Juveniles responded to the handling with rapid escape behavior and subsequent immobility. Latency to restart activity (time spent immobile) was used as a proxy for risk‐taking with individuals restarting normal activity quickly seen as risk‐takers (Urszán, Garamszegi, et al., 2015). Juveniles' behavior was video‐recorded for 10 min. Out of the 136 assays, individuals remained immobile during the 10‐min observation period in only six cases (three individuals). These observations received maximal score (600 s). We note that the video evaluation was not blind regarding the test animals' identity, because the identification number of the animals was visible in the video recordings. However, it does not pose a problem since the subjectivity in our methods was negligible.

### 2.5 Locomotor performance

After the behavioral tests, climbing speed of offspring was assessed as a measure of locomotor performance (see Cadby et al., 2010; Wapstra, 2000). As *I. cyreni* is a highly specified, scansorial, rock‐dwelling species (Martín & López, 1999; Martín & Salvador, 1997; Monasterio, Salvador, & Díaz, 2010), climbing speed is more relevant for performance in this species than sprint speed on a horizontal plane. In these tests, a predator attack was simulated by tapping lizards close to the tail with a brush to stimulate them to run along a racetrack (0.5 m × 0.15 m; length, width, respectively) tilted to an angle of 90° and covered with sandpaper (see Gomes, Carretero, & Kaliontzopoulou, 2016; Ortega, López, & Martín, 2014). Tests were performed between 12:00 and 15:00 p.m. (UTC + 02.00) when lizards were fully active (see Aragón, López, & Martín, 2004; Martín & Salvador, 1995; Pérez‐Mellado et al., 1988). Each lizard was tested two times during two consecutive days (22 and 23 September). Before climbing trials, the animals were kept at 30°C for one hour to allow them to reach optimal temperatures (Aguado & Braña, 2014). Tests were recorded with a digital video camera (Panasonic HC‐V160, Panasonic Co.) at 25 fps. The runs were graded on a scale of 1–5. Runs with 1 representing individuals that refused to run and 5 representing individuals that run through the entire track without stops (see Collins, Self, Anderson, & McBrayer, 2013). Only runs scored 4 and 5 were considered for speed tests (see van Berkum & Tsuji, 1987; Vanhooydonck, Van Damme, & Aerts, 2001). Based on this criterion, runs from four individuals of control mothers (two placeboes and two corticosterone) were discarded. To extract speed measures, video records were analyzed with the software TRACKER v.4.97 (Open Source Physics). Maximum climbing speed for each individual was determined as the highest speed (cm/s) recorded within any two successive frames in any trial (Gomes, Carretero, & Kaliontzopoulou, 2017; Martín & López, 1995).

### 2.6 Statistical analyses

To test whether our treatments affected offspring SVL, BW, and climbing speed, we ran three separate linear mixed models (LMMs). In the first LMM, we used SVL as response variable, while maternal vitamin supplementation, corticosterone treatment, and their interaction were added as fixed factors. Mother identity was added as a random factor to account for the nonindependence of individuals within a single family. The second model on BW was built in the same manner; however, to control for size, we added SVL as a covariate. In the third model, climbing speed was our response variable, the treatments and their interaction were added as fixed factors and both SVL and BW were included as covariates to control for the effect of size and condition.

Behavioral measures were square‐root‐transformed in order to achieve normal distributions of the model residuals. We used repeatability of behavior (i.e., strength of animal personality) as the first estimate of behavioral variation. To estimate repeatability of the behavioral traits in the pooled sample and treatment groups, we ran LMMs separately with the behavioral variable of interest as dependent variable and individual as random factor using restricted maximum‐likelihood estimation in the lme4 package (Bates, Mächler, Bolker, & Walker, 2015). Confidence intervals were calculated by nonparametric bootstrapping, while significance is provided by random permutation (also called randomization test), both sampled at each 1,000th iteration.

Behavioral type (individual mean behavior) was used as the second measure of behavioral variation. We ran LMMs on the behaviors separately to test whether treatments affected behavioral type. In these models, the different behavioral traits were our response variables, treatments and their interactions were added as fixed effects, while mother identity and individual were random factors. We added z‐transformed order of trials (hereafter: time) both as a single fixed effect and as a random slope (i.e., in interaction with individual) to the models to test for habituation on the group and individual levels directly.

Behavioral predictability was the third measure of behavioral variation. To characterize behavioral predictability, we calculated residual individual standard deviation scores (riSD; Chang et al., 2017; Lichtenstein et al., 2017; Stamps et al., 2012). To this end, we built simplified LMMs for each behavioral trait separately, with behavior as response variable, time as fixed and individual (random intercept) and the individual × time interaction (random slopes) as random effects. Predicted values for each individual were extracted from the models and compared with the observed values to get residual values for each risk‐taking observation. We calculated riSD scores following the method of Stamps et al. (2012), however, based on N observations, rather than N−1, as this provides a more conservative approach to detect significant differences in riSDs (see Briffa, 2013). We performed similar LMMs to those of the behavioral types, but with riSD as response variable, and only mother identity as random factor.

Backward elimination was applied in order to simplify our models by removing nonsignificant effects from LMMs provided by the package lmerTest (Kuznetsova, Brockhoff, & Christensen, 2016). We also report the proportion of explained variance by the fixed factors (marginal *R^2^*) and by both fixed and random factors (conditional *R* ^2^) available in the MuMIn package (Bartoń, 2016). These values represent goodness‐of‐fit measures of GLMMs similar to the *R* ^2^ value of generalized linear models (Nakagawa, Johnson, & Schielzeth, 2017; Nakagawa & Schielzeth, 2013). All analyses were performed using the R statistical environment (R Developmental Core Team, 2018).

### 2.7 Ethical approval

All applicable international, national, and/or institutional guidelines for the care and use of animals were followed. The experiment was performed under license (permit number: 10/056780.9/16) from the Environmental Agency of Madrid Government (“Consejería de Medio Ambiente de la Comunidad de Madrid,” Spain).

## 3 RESULTS

### 3.1 Offspring quality

**Figure 2:**
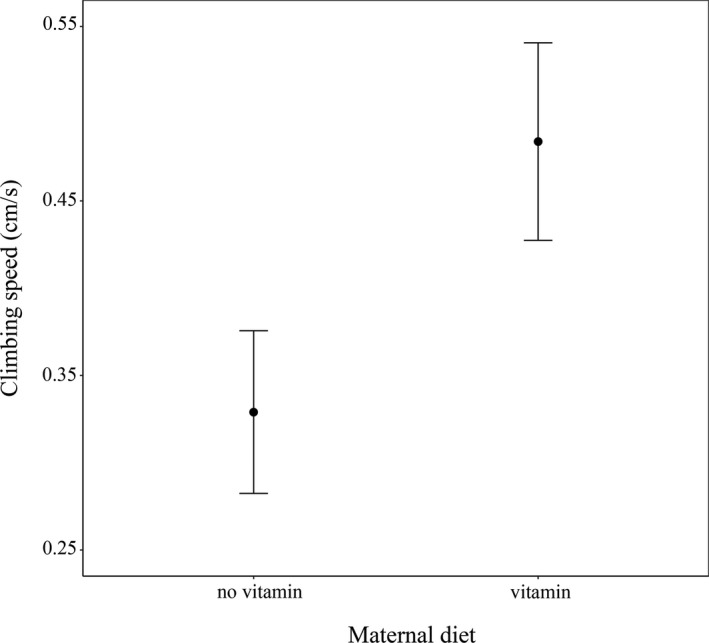
Differences in climbing speed of juvenile *Iberolacerta cyreni* induced by maternal vitamin D_3_ supplementation. Least square means and standard errors are shown

According to our LMMs, none of our treatments affected offspring SVL or BW (see Table 1). On the other hand, the third model revealed an effect of maternal vitamin treatment on offspring's climbing speed (*F*_1,17.25_ = 5.24, *p* = .035; Figure 2): juveniles from vitamin‐supplemented mothers climbed 42.4% faster. Corticosterone had no effect on climbing speed, neither alone nor in interaction with vitamin supplementation (see Table 1). The family effect was significant (*χ* ^2^ = 3.9, *df* = 1, *p* = .048). We found no effect of SVL or BW on climbing speed (Table 1).

**Table 1:** Results of LMMs on morphology of juvenile *Iberolacerta cyreni*

Dependent variable	Fixed effects	*F *(*df*_1_, *df*_2_)	*p*
SVL	Corticosterone (C)	0.95 (1; 17.32)	.34
	Maternal diet (M)	1.17 (1; 30.21)	.29
	C × M	0.98 (1; 16.87)	.33
BW	C	0.16 (1; 18.79)	.69
	M	0.001 (1; 30.99)	.99
	C × M	0.58 (1; 17.55)	.45
	SVL	3.79 (1; 32.94)	.06
Climbing speed	C	0.21 (1; 16.15)	.66
	**M**	**5.24 (1; 17.25)**	**.035**
	C × M	0.16 (1; 16.22)	.69
	SVL	0.19 (1; 31.68)	.66
	BW	0.14 (1; 29.55)	.71

Note

*F* statistics (numerator and denominator *df* in parentheses) and *p* values are shown. Significant effects are in bold font.

### 3.2 Behavioral repeatability

Random permutation indicated moderate repeatability for all behavioral traits in the pooled sample (Table 2). As random permutation gives robust measures of statistical significance for Gaussian data (Nakagawa & Schielzeth, 2010), we considered activity personality being significant, in all treatment groups, with estimates showing moderate/high repeatability (0.31 < *R* < 0.68; see Table 2). On the other hand, confidence intervals for activity repeatability estimates highly overlapped between treatment groups and with that of the pooled sample; thus, repeatability estimates did not differ between treatment groups and the whole sample. In the case of shelter use, repeatability was significant only in the vitamin‐supplemented × corticosterone‐treated group, where the estimate indicates particularly high repeatability (*R* = 0.9; Table 2). In risk‐taking, corticosterone supplementation resulted in the lack of significant repeatability, while repeatabilities in the corticosterone control groups can be considered moderate to high (0.4 < *R* < 0.57; see Table 2).

**Table 2:** Repeatability estimates for activity, shelter use, and risk‐taking in juvenile *Iberolacerta cyreni* in the pooled sample (all) and in the different treatment groups (CORT corticosterone, NoCORT corticosterone control, VIT vitamin D_3_ supplemented mothers, NoVIT control mothers)

ALL (*N* = 34)	VIT/CORT (*N* = 7)	NoVIT/CORT (*N* = 10)	VIT/NoCORT (*N* = 7)	NoVIT/NoCORT (*N* = 10)
Activity				
***R* = 0.415**	***R* = 0.363**	***R* = 0.305**	***R* = 0.676**	***R* = 0.326**
***p* < .001**	***p* = .02**	***p* = .011**	***p* = .001**	***p* = .006**
**CI = 0.21–0.58**	**CI = 0–0.7**	**CI = 0–0.61**	**CI = 0.12–0.87**	**CI = 0–0.61**
Shelter use				
***R* = 0.28**	***R* = 0.9**	*R* = 0.08	*R* = 0.12	*R* = 0.1
***p* < .001**	***p* < .001**	*p* = .118	*p* = .099	*p* = .16
**CI = 0.07–0.44**	**CI = 0.63–0.96**	CI = 0–0.38	CI = 0–0.5	CI = 0–0.42
Risk‐taking				
***R* = 0.332**	*R* = 0.219	*R* < 0.001	***R* = 0.396**	***R* = 0.569**
***p* < .001**	*p* = .055	*p* > .99	***p* = .012**	***p* < .001**
**CI = 0.13–0.5**	CI = 0–0.57	CI = 0–0.299	**CI = 0–0.71**	**CI = 0.18–0.8**

Note

Estimates are based on linear mixed models (LMMs). Repeatabilities (*R*) and 95% confidence intervals (CI) are shown. Significance (*p*) estimates are based on randomization tests. Significant repeatabilities are in bold font.

### 3.3 Behavioral type and predictability

LMMs revealed no effects of any treatments on behavioral types. However, we found significant habituation in activity (*F*_1,102_ = 5.2, *p* = .02): Juveniles became more active by time, although individual trends did not differ (*χ* ^2^ = 0.19, *df* = 1, *p* = .66). In contrast, there was no sign of habituation in shelter use or risk‐taking (shelter use: *F*_1,102_ = 2.1, *p* = .15; risk‐taking: *F*_1,101.9_ = 1.26, *p* = .26), and there was no difference between individual trends (shelter use: *χ* ^2^ < 0.001, *df* = 1, *p* > .99; risk‐taking: *χ* ^2^ = 0.76, *df* = 1, *p* = .39). The fixed effects explained 5.4% (activity), 6.3% (shelter use), and 3.6% (risk‐taking) of the total variance, while the full models 44.4% (activity), 25.2% (shelter use), and 46.8% (risk‐taking), which can be seen as sufficient explanatory power for behavioral variables. For remaining nonsignificant effects, see Table 3.

**Table 3:** Results of LMMs on activity, shelter use, and risk‐taking behavioral types of juvenile *Iberolacerta cyreni*

Fixed effects	Activity		Shelter use		Risk‐taking	
	*F* (*df*_1_, *df*_2_)	*p*	*F* (*df*_1_, *df*_2_)	*p*	*F* (*df*_1_, *df*_2_)	*p*
Corticosterone	0.084 (1, 34)	.37	0.91 (1, 17)	.36	0.005 (1, 35.41)	.94
Maternal diet	1.21 (1, 34)	.28	3.24 (1, 17)	.089	1.85 (1, 35.24)	.18
Corticosterone × Maternal diet	0.004 (1, 34)	.95	0.28 (1, 17)	.6	0.01 (1, 35.21.7)	.92
Time	**5.2 (1, 102)**	**.02**	2.1 (1, 102)	.15	1.26 (1, 101.9)	.26

Note

*F* statistics (numerator and denominator *df* in parentheses) and *p* values are shown. Significant effects are in bold font.

**Figure 3:**
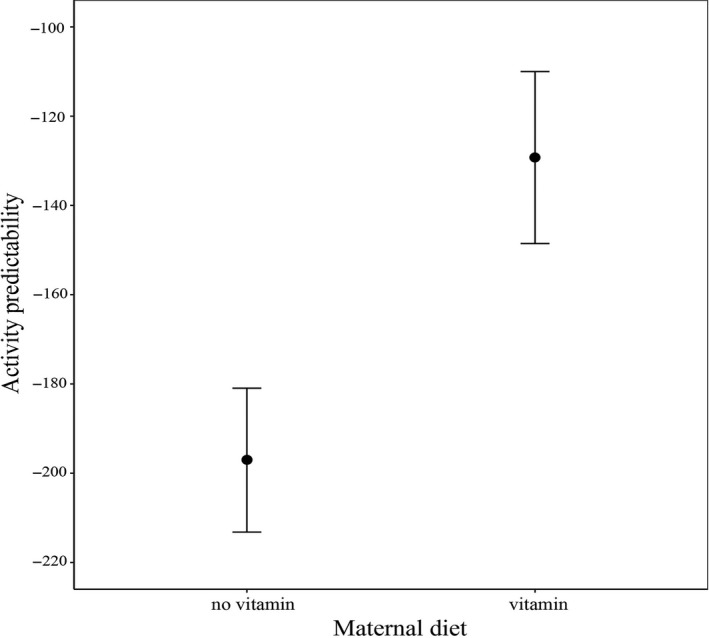
Differences in activity predictability of juvenile *Iberolacerta cyreni* induced by maternal vitamin D_3_ supplementation. Predictability (riSD) values are multiplied by −1 for straightforward interpretation; hence, small values translate to low predictability in the figure. Least square means and standard errors are shown

LMMs revealed a significant vitamin D_3_ supplementation effect on activity predictability (*F*_1,34_ = 7.74, *p* = .009): Juveniles from vitamin‐supplemented mothers were more predictable (Figure 3). Treatments did not affect shelter use or risk‐taking predictability (see Table 4.). The fixed effects explained 25.9% (activity), 18.7% (risk‐taking), and 0.7% (shelter use) of the total variance, while the full models explained 25.9% (activity), 53.9% (risk‐taking), and 30.5% (shelter use), which can be seen as sufficient explanatory power for behavioral variables. For the nonsignificant effects, see Table 4.

**Table 4:** Results of LMMs on activity, shelter use, and risk‐taking behavioral predictability of juvenile *Iberolacerta cyreni*

Fixed effects	Activity		Shelter use		Risk‐taking	
	*F* (*df*_1_, *df*_2_)	*p*	*F* (*df*_1_, *df*_2_)	*p*	*F* (*df*_1_, *df*_2_)	*p*
Corticosterone	0.59 (1, 33.99)	.45	0.05 (1, 16.95)	.83	3.18 (1, 16.99)	.093
Maternal diet	**7.74 (1, 34.004)**	**.009**	0.14 (1, 17)	.71	1.87 (1, 17)	.19
Corticosterone × Maternal diet	2.48 (1, 34.008)	.12	0.04 (1, 17.09)	.84	3.99 (1, 16.99)	.62

Note

*F* statistics (numerator and denominator *df* in parentheses) and *p* values are shown. Significant effects are in bold font.

## 4 DISCUSSION

Here, we manipulated two biologically relevant factors (composition of maternal diet and corticosterone levels) that are expected to have a profound effect both on offspring permanent‐ and short‐term state and, thus, on behavioral consistency. Based on our results, maternal diet affected offspring climbing speed positively; moreover, we found significant treatment effects on different levels of juveniles' behavioral consistency, suggesting that individual quality can modify the effect of corticosterone in “switching” individual behavioral variation on/off. We discuss our results in light of the evolutionary consequences of the presence/absence of individual differences in highly flexible phenotypic traits.

### 4.1 Offspring quality

Somewhat surprisingly, corticosterone treatment did not affect offspring climbing speed; however, we found a clear effect of maternal vitamin treatment: Juveniles from vitamin D_3_ supplemented mothers had higher climbing speed (42.4% increase) than conspecifics from control mothers. Locomotor performance is known to potentially increase the ability to escape from predators (Bauwens, Garland, Castilla, & Van Damme, 1995; Irschick & Garland, 2001), foraging efficacy (Husak, Ferguson, & Lovern, 2016; Irschick, Meyers, Husak, & Le Galliard, 2008), and social dominance status in lizards (Clobert et al., 2000; Garland, Hankins, & Huey, 1990; López & Martín, 2002), which makes it a suitable proxy of individual fitness (Irschick et al., 2005; Winchell et al., 2018). Previous data on reptiles indicate the importance of maternal investment on offspring's traits linked to individual state (Meylan et al., 2002; Munch et al., 2018; Wapstra, 2000); however, there are contradictory results as well (Ensminger et al., 2018; Warner, Lovern, & Shine, 2008). Our results provide, to our knowledge for the first time, empirical data that the amount of vitamin D_3_ in maternal diet increases offspring locomotor performance in reptiles. Vitamin D_3_ is implied to be a hormonally active vitamin essential in the development of organs and maintenance of calcium homeostasis in amniotes (Karsten, Ferguson, Chen, & Holick, 2009; Laing & Fraser, 1999); nevertheless, its exact role during reproduction and embryogenesis is much less understood. Recent empirical work on rodents indicates that maternal vitamin D_3_ intake affects offspring health and bone structure positively (Lanham et al., 2013; Luk, Torrealday, Perry, & Pal, 2012). However, since the exact downstream mechanisms likely differ between mammals and reptiles, direct comparison between our and these previous findings is not possible. Nevertheless, our results suggest that vitamin D_3_ is indeed an important factor of embryogenesis and an important maternal investment that potentially affects offspring fitness.

Families differed significantly in climbing speed. Even if we assume that fathers were different among clutches, as we had F1 generation, maternal or cross‐generational environmental effects cannot be ruled out, and thus, the additive genetic contribution cannot be proven (Lynch & Walsh, 1998).

### 4.2 Behavioral repeatability

There were no evident treatment effects on the repeatability of activity. Repeatability of shelter use was moderate in the pooled sample, while it was significant only in the vitamin‐supplemented × corticosterone‐treated treatment group, consistency being particularly strong in this treatment (see Bell et al., 2009 for reference). This type of challenge‐triggered consistency is contrary to previous finding on wild‐caught adults of *I. cyreni*, where shelter use personality (estimated similarly to the present study) was detected only under optimal conditions (Horváth, Martín, et al., 2017). In the present approach, shelter use describes “general” (as opposed to movement) activity, as individuals spending more time hiding were regarded as less active (Koolhaas et al., 1999; Réale et al., 2007). Regarding the exact mechanisms causing the emergence of personality in this group (or the collapse in the others), we can only speculate. Perhaps different tactics in exposure to predators in favor of foraging under stress could only be chosen by individuals with high climbing speed and thus good escape abilities. Risk‐taking proved to be moderately repeatable in the pooled sample, and moderate–high in the corticosterone control groups, while repeatability was negligible in the corticosterone‐treated groups. This pattern is somewhat in line with our previous findings on repeatability of risk‐taking in adult male *I. cyreni*: Individuals under suboptimal conditions tend to shift their behavior toward a general strategy, resulting in decreased interindividual variation (Horváth, Martín, et al., 2017). The fact that under certain circumstances animal personality can quickly fade implies that drastic changes in behavioral strategy could occur not just among years, but among seasons within the same year (see Garamszegi et al., 2015). More importantly, quick cessation of behavioral variation may have serious evolutionary consequences, since at least initial response to selection depends on existing between‐individual phenotypic variation (Barton & Partridge, 2000; Farine, Montiglio, & Spiegel, 2015). Confidence intervals of repeatabilities in the treatment groups highly overlapped with that of the pooled sample in all studied behaviors, indicating no significant difference between repeatability estimates of the treatment groups and the pooled sample. These results suggest that lowered variation in individual states does not weaken behavioral consistency, similarly to what we find about the effect of environmental variation in adult males of *I. cyreni* and *L. viridis* (Horváth, Martín, et al., 2017; Horváth, Mészáros, et al., 2017; respectively).

We are aware that the sample size per vitamin D_3_ × corticosterone treatment groups is somewhat low, making the interpretation of lack of significant repeatabilities questionable. However, we must note that the highest repeatability estimate with no statistical support was 0.22 (*p* = .055), which can already be seen as low repeatability (see Bell et al., 2009), and the rest of nonsignificant estimates fell below 0.16. Hence, we think that the pattern we present regarding the presence/absence of behavioral repeatability (a test for animal personality) is robust. Although we found significant habituation in activity (juveniles became more active by time), consistent between‐individual differences were detected irrespective of general trends of habituation. Hence, we are convinced that our assays give a good estimate of individual behavioral differences (Biro, 2012).

### 4.3 Behavioral type and predictability

Regarding behavioral types, our expectation was that juveniles from vitamin‐supplemented mothers and under low levels of exogenous corticosterone behave bolder (i.e., being more active, risk‐prone and spend less time hiding), either because of their superior quality or less challenging levels of physiological stress. To the contrary, none of our treatments affected behavioral types significantly. This is interesting because it shows that individual quality can affect behavioral strategies (presence of personality, see above; individual behavioral predictability, see below) without affecting group‐level mean behavior.

In contrast to the lack of significant treatment effects on behavioral types, we found significant effect of maternal diet on activity predictability: Juveniles from control mothers were less predictable. Quick growth at the juvenile stage is a good strategy to reach maturity early, and foraging activity is a key factor of maximizing energy intake (Biro & Stamps, 2008; Brodin & Johansson, 2004). However, increased behavioral activity results in higher exposure to predators. Because of their lower quality (i.e., poorer climbing speed), juveniles from control mothers potentially are under higher predation risk, as they cannot flee as quickly as their conspecifics. Expressing behavior in an unpredictable way might be an effective antipredator strategy, as predators often rely on predictable patterns in prey locomotion (Briffa, 2013; Highcock & Carter, 2014; Richardson, Dickinson, Burman, Pike, & Pike, 2018). Hence, this pattern is somewhat in line with the predictions of state‐dependent safety principle (Dosmann, Brooks, & Mateo, 2014; Luttbeg & Sih, 2010; Sih et al., 2015), where individuals with low climbing speed suffer higher predation risk while being active and should thus be less predictable (but see Urszán et al., 2018).

## 5 CONCLUSIONS

Taken together, our results support the notion that differences in permanent individual state (i.e., climbing speed) induced by maternal diet can affect the expression of between‐individual behavioral variation, that is, animal personality. In some of the cases, the effects depended on exposure to exogenous corticosterone. Hence, our results indicate that maternal effects can have a major role in the emergence of behavioral consistency. This is not only important in understanding the proximate mechanisms behind the development of animal personality but has a broader evolutionary significance too. Since our treatments could induce or erode consistent behavioral variation among juvenile lizards, and considering that natural selection can only operate on existing between‐individual phenotypic variation, short‐term variation in environmental conditions and individual state might—at least temporarily—release a population from natural selection acting on behavioral variation (see also Horváth, Martín, et al., 2017; Horváth, Mészáros, et al., 2017).

## CONFLICT OF INTEREST

None declared.

## AUTHOR CONTRIBUTION

GeH, GR‐R, JM, PL, and GH designed the study; GeH and GR‐R collected data and performed the experiments; GeH analyzed the data with the contribution of GR‐R and GH; GeH, and GH wrote the manuscript with the substantial contribution of GR‐R, JM, and PL; all authors reviewed the manuscript and gave final approval for publication.

### Open Research Badges

This article has earned an https://openscience.com Badge for making publicly available the digitally‐shareable data necessary to reproduce the reported results. The data is available at http://doi.org/10.5061/dryad.sqv9s4n0g.

## Data Availability

Data from this study are archived in the public archive Dryad (http://datadryad.org) at the http://doi.org/10.5061/dryad.sqv9s4n0g (Horváth, Rodríguez‐Ruiz, Martín, López, & Herczeg, 2019).
